# Impact of fast advancement and early fortification of enteral feedings in preterm infants, a retrospective, observational study

**DOI:** 10.3389/fped.2025.1544381

**Published:** 2025-09-19

**Authors:** Enrique Gomez-Pomar, Holly Clarke, Jamie Adams

**Affiliations:** ^1^ Division of Neonatology, Department of Pediatrics, University of Kentucky, Lexington, KY, United States; ^2^ Neonatology, St Bernards Regional Medical Center, Jonesboro, AR, United States; ^3^ Astarte Medical, Yardley, PA, United States

**Keywords:** neonatal nutrition, preterm growth outcomes, preterm enteral nutrition, preterm sepsis, premature infants, preterm growth failure

## Abstract

We aimed to evaluate the outcomes and growth of preterm infants at a Neonatal Intensive Care Unit that focuses on early initiation, fast advancement, and early fortification of enteral feeds.

**Methods:**

Retrospective study conducted at a community hospital level III NICU. Extensive feeding data and outcomes were collected by utilizing a NutritionIQ software application, NICUtrition®. Infants born between 26- and 34-weeks gestational age were included, whereas infants with congenital defects, deceased or with incomplete data were excluded. Frequency and descriptive statistical analysis were conducted using chi-square and Fisher's exact test. Unadjusted odds ratios were computed for categorical variables and general linear models were conducted to adjust for covariates (birth weight and gestational age) in sensitivity analyses.

**Results:**

A total of 297 preterm infants were included. On average, infants reached target enteral feeds of at least 120cc/kg/day and received fortification by day 6 of life. Achievement of target enteral feeds within the first week of life was associated with improved delta z-scores for weight and length as well as significantly less rates of sepsis. Infants that achieved target enteral feedings and fortification during the first week of life were associated with significantly improved delta z-scores for weight, length, and head circumference. Contrary to expectations, the use of Mother's Own Milk alone was not associated with improved outcomes.

**Conclusions:**

This study highlights the association of early initiation, faster advancement and fortification of enteral feedings on preterm infants. These interventions improved growth metrics (weight, head circumference, and length z-scores) and were associated with decreased prevalence of sepsis.

## Introduction

Infants born prematurely are at risk of developing extrauterine growth failure and becoming malnourished, a significant issue affecting up to 40% of all infants discharged from the Neonatal Intensive Care Unit (NICU) (1). After birth, infants experience a sudden interruption in their nutritional supply, entering a catabolic state that becomes more pronounced with increasing prematurity of the infant. This nutritional deficit accumulates over time, causing significant adverse effects that persist well after NICU discharge (2–4). Parenteral nutrition (PN) is a way to ameliorate protein and nutritional deficits and should be started as soon as possible; yet, enteral feeds as well as the use of human milk remain the gold standard of neonatal nutrition (5, 6). Unfortified or plain enteral feeds of human milk, do not provide sufficient nutrients to supply the superior nutritional needs of preterm infants, thus the need for fortification (7).

Feeding practices have evolved throughout the years (8). Current evidence supports starting enteral feeds as soon as possible, advancing as soon and as fast as the infant tolerates, fortifying feeds, and ultimately achieving a total fluid volume of at least 150–160 cc/kg/day (7, 9–11). Despite this evidence, a current lack of consensus and knowledge of best practice for the feeding of preterm infants continues to lead to significant variations related to the speed of advancement or fortification of enteral feedings as well as the unfounded concern that early and fast advancement of enteral feedings may lead to adverse effects, especially in the most vulnerable infants (10, 12–17).

In this retrospective study, we aim to evaluate how early achievement of target enteral feeding volumes as well as early fortification influences neonatal outcomes in preterm infants. We evaluate the resulting growth outcomes, PN use, rates of necrotizing enterocolitis (NEC), sepsis, bronchopulmonary dysplasia (BPD), intraventricular hemorrhage (IVH) and retinopathy of prematurity (ROP).

## Methods

Retrospective study conducted in a level III NICU located at a community hospital treating preterm infants ≥26 weeks GA and birth weight ≥750 gm. This study was approved by the hospital's Institutional Review Board. By utilizing a NutritionIQ software application, NICUtrition®, that pulled information directly from the Electronic Medical Record (EMR); extensive growth, feeding and nutritional data was extracted, along with the reported outcomes for all preterm infants born at ≤34 weeks GA from 2016 to 2023. The data was reviewed for errors with exclusion of any infants with incomplete data, those who were deceased, transferred to an outside hospital, or who had congenital defects. The data was also reviewed and compared to the EMR to ensure accuracy and when inaccuracies were encountered, the infant was removed from the database.

Weight was analyzed at 36 weeks corrected GA (cGA) and at discharge; z-scores were calculated for weight, head circumference, and length (18). Target enteral feeding volume was defined as the day when the infant received a feeding volume of at least 120 cc/kg/day. The diagnoses of culture positive sepsis, NEC (any category), IVH (any grade), BPD (any category) and ROP (any stage) were extracted from the charts as diagnosis codes recorded by the providers. Of note, BPD was initially defined as the need for oxygen at 36 weeks cGA; however, in recent years the National Institute of Child Health and Human Development (NICHD) definition was adopted (19).

### Unit feeding practices

Since 2016, the standard of care has been to start trophic feedings within 24 h of birth. Infants less than 34 weeks GA are fed with human milk, either Mother's Own Milk (MOM) or Donor Breast Milk (DBM). Enteral feeds are initiated at 20–30 cc/kg/day and advanced every 12 h by ∼15 cc/kg, per infant's tolerance, until a total feeding volume of 150–160 cc/kg/day is achieved. While advancing enteral feeds, PN is provided to account for the remaining fluid volume needs according to the infant's GA and Day of Life (DOL). Normally, PN is provided via a central venous line (most commonly Umbilical Venous Catheters) and discontinued once the infant reaches a target enteral feeding volume of 120–130 cc/kg/day.

Administration of enteral feeds is via nasogastric or orogastric tube at 3 h intervals. Feeds are initially administered via gravity until the volume is above 10 ml and thereafter put on a feeding pump to be given over 30 min. If the infant develops significant spitting between feeds (after ruling out other causes of feeding intolerance), the time is increased by 15 min intervals up to a maximum feeding time of 150 min. If episodes of increased spitting/vomiting continue after achieving max feeding time, feeds are instead given continuously by a transpyloric tube, a rare occurrence of less than 1 infant per year. Once the infant is tolerating enteral feeds well with minimal or no spitting/vomiting, the feeding times are progressively compressed to a goal of 30 minutes.

Fortification of enteral feeds is provided, via standard mode, to all preterm infants once total enteral feed volume is ∼100 cc/kg/day by using a multi-nutrient liquid Human Milk Fortifier (HMF) (20). During the study period, HMF use was from either Enfamil Liquid HMF High Protein (Mead Johnson) or Similac HMF Extensively Hydrolyzed Protein Concentrated Liquid (Abbott Nutrition), differing only due to product availability or for an intolerance by the infant. Initially, fortification was advanced to 22 kcal/oz and then 24 kcal/oz; though, most recently, fortification starts at 24 kcal/oz. Early fortification was defined as receiving any multi-nutrient fortifier during the first week of life. Once the infant is within days of discharge, fortification with HMF is transitioned to powder preterm formula. If MOM is unavailable, DBM is continued until around 34–35 weeks GA when feedings are fully transitioned to preterm formula.

Enteral feeds are contraindicated for infants with congenital gastrointestinal malformations or those who need to be transferred for surgical procedures or cooling (not relevant to current study population as they were excluded). Infants who are acutely ill (hemodynamic or respiratory instability) are not fed for a period of 6–12 h after birth; however, enteral feeds are recommended to begin as soon as the infant is hemodynamically stable and/or metabolic acidosis has resolved. If an infant is hemodynamically stable yet receiving blood products, the feeds are held only during the transfusion time (one feeding) and subsequently restarted at full volume after the transfusion. Enteral feeds are provided to infants with umbilical arterial and venous catheters, as well as infants who are intubated or with chest tubes. Infants receiving vasoactive medications and/or steroids are fed on a case-by-case basis. Feeds are held if there is a concern for the development of NEC but are restarted once there is resolution or no progression of the signs of NEC (abdominal distension, cardio-respiratory instability, or radiographic signs). Enteral feeds are restarted unfortified, at half full enteral feeding volume and quickly advanced to full-fortified enteral feeding volumes within the next 24 h.

Oral readiness is primarily assessed by bedside nursing. Previously, infants who showed oral feeding cues started trials of oral feedings and advanced once demonstrating progressive cues or successful oral attempts. Since 2022, speech therapists have been added to the NICU team and start providing milk drops to infants as soon as they are hemodynamically stable (21). Evaluation of oral readiness occurs daily after 32 weeks cGA via an objective tool with oral attempts starting once the infant achieves a compatible score. The number of total daily oral attempts increases as the infant demonstrates successful completion. Oral feedings are provided to infants with respiratory rates below 70 breaths per minute that are not on respiratory support, on respiratory support via Humidified High Flow Nasal Cannula with a flow less than 3 L/min, and to those on Low Flow Nasal Cannula.

Despite the feeding recommendations in the unit, there is some degree of variability in feeding practices due to provider preferences and individual neonatal variations in feeding tolerances. Length of stay was calculated from the day of admission to the day of discharge from the hospital and discharge readiness is assessed based on infants’ weight (≥1,850 gm), ability to tolerate full enteral Ad Libitum feeds with good volume intake, adequate average weight gain (last 7 days) and temperature and respiratory stability.

### Statistical analysis

Frequency and descriptive statistics were used to summarize demographic and clinical characteristics of the sample. For the between-subjects comparisons of categorical variables, either the chi-square test or Fisher's exact test was used as appropriate, and the results were reported with frequencies and percentages to inform upon the inferential findings. For a subset of categorical variables, unadjusted odds ratios (OR) with 95% confidence intervals (CI) were calculated. Due to violations of the assumptions of normality and homogeneity of variance, non-parametric Mann–Whitney *U* tests were employed to compare groups on continuous variables. Corresponding medians and interquartile ranges (IQRs) were reported. General Linear Models (GLM) were conducted to adjust for covariates (birth weight and gestational age) in sensitivity analyses, and adjusted means and 95% CIs were generated for the independent groups. A two-sided alpha level of 0.05 was used to determine statistical significance and all analyses were performed using SPSS Version 29 (IBM Corp., Armonk, NY).

## Results

A total of 297 preterm infants were included in the study; overall characteristics are described in Table 1. Classification of the data was arranged into those who achieved target enteral feeding volumes during the first week of life vs. those who did not (Table 2). Overall, infants that were fed faster were significantly older and bigger at birth with subsequent shorter NICU stays. They received significantly less days of PN and had significantly improved delta z-scores for weight and length compared to their counterparts. After controlling for GA and birth weight, infants who took longer to achieve target feeding volume had 23.57 (95% CI 4.40–126.36, *p* < 0.001) higher odds of being diagnosed with sepsis during the hospital stay.

**Table 1:** Preterm infants characteristics and outcomes.

Variable	Value (IQR)
# of infants	297
Birth gestational age	32.0 (30.0–33.0)
Birth weight (g)	1,660 (1,380–1,970)
Birth weight percentile	45.07% (24.64%–64.55%)
Birth weight z-score	0.04 (−0.57–0.63)
Birth head circumference (cm)	29.0 (27.0–30.0)
Birth head circumference z-score	−0.20 (−0.88–0.43)
Birth length (cm)	42.5 (40.0–44.5)
Birth length z-score	0.30 (−0.36–0.98)
First enteral feed in days	1 (0–1)
First target enteral feed volume in days	6 (4–7)
First fortification in days	6 (4–9)
Return to birth weight in days	12.5 (9.5–15)
Parenteral nutrition in days	5 (3–7)
Growth velocity in g/kg/day	22.67 (18.21–26.15)
Length of stay in days	33 (23–48)
Human milk (% total volume given)	70% (38%–95%)
Mother's milk (% total volume given)	26% (0%–85%)
Weight 36 weeks cGA (g)	2,326 (2,064–2,638)
Weight 36 weeks cGA z-score	−1.15 (−1.69 to −0.42)
Discharge weight (g)	2,419 (2,134–2,883)
Discharge weight percentile	26.10% (14.28%–49.75%)
Delta z-score for weight at discharge	−0.64 (−1.07 to −0.01)
Discharge head circumference (cm)	32.0 (31.0–34.0)
Discharge head circumference z-score	−0.34 (−0.93–0.29)
Delta z-score for head circumference	−0.20 (−0.76–0.42)
Discharge length (cm)	46.5 (44.5–49.0)
Discharge length z-score	−0.34 (−0.96–0.43)
Delta z-score for length	−0.58 (−1.23–0.08)
Necrotizing enterocolitis rate	0.3% (−0.3%–0.1%)
Intraventricular hemorrhage rate	4.0% (2.3%–6.9%)
Bronchopulmonary dysplasia rate	7.4% (4.9%–11%)
Retinopathy of prematurity rate	30.0% (25.0%–35%)
Sepsis rate	4.4% (2.6%–7.3%)

Values expressed in frequency (%) or medians and IQR. Rates calculated from cases/study population.

**Table 2:** Preterm infants that achieved target enteral feeding volume during the first week of life.

Variable	<8 days	≥8 days	*p*-value
# of Infants	253	42	–
Birth gestational age	32 (31–33)	28.5 (27–30)	<0.001
Birth weight (g)	1,730 (1,500–2,010)	1,175 (1,000–1,365)	<0.001
Birth weight percentile	45.07% (24.82%–64.43%)	44.38% (21.89%–65.10%)	0.99
Birth weight z-score	0.05 (−0.53 to 0.63)	−0.04 (−0.64 to 0.61)	0.81
Birth head circumference (cm)	29 (28.0–30.0)	26 (24.0–27.0)	<0.001
Birth head circumference z-score	−0.14 (−0.81 to 0.47)	−0.29 (−1.18 to 0.37)	0.14
Birth Length (cm)	43.0 (40.6–45.0)	38.1 (36.0–41.3)	<0.001
Birth length z-score	0.25 (−0.36 to 1.01)	0.44 (−0.38 to 0.73)	0.91
First enteral feed in days (IQR)	1 (0–1)	1 (1–1)	<0.001
First target enteral feed in days (IQR)	5 (4–6)	10 (9–11)	<0.001
First fortification in days (IQR)	6 (4–8)	10.5 (9–12)	<0.001
Return to birth weight in days (IQR)	13 (10–15)	11 (9–16)	0.85
Parenteral nutrition in days (IQR)	5 (1–6)	10 (9–16)	<0.001
Growth Velocity in g/kg/day (IQR)	22.67 (17.75–26.74)	22.65 (19.24–25.81)	0.66
Length of stay in days (IQR)	31.0 (22–44)	57.5 (43–71)	<0.001
Human milk (% total volume given)	70% (36%–97%)	69.5% (51%–86%)	0.89
Mother's milk (% total volume given)	27% (0%–86%)	23% (1%–71%)	0.70
Weight 36 weeks cGA (g)	2,342.5 (2,087.5–2,640.5)	2,182.0 (2,025–2,419)	0.10
Weight 36 weeks cGA z-score	−1.09 (−1.62 to −0.37)	−1.50 (−1.82 to −0.97)	0.051
Discharge weight (g)	2,430 (2,148–2,873)	2,293 (20.97–3,126)	0.83
Discharge weight percentile	27.42% (15.29%–51.04%)	18.50% (10.00%–33.92%)	0.046
Discharge weight z-score	−0.60 (−1.02 to 0.03)	−0.90 (−1.28 to −0.42)	0.046
Delta z-score for weight	−0.65 (−0.94 to −0.33)	−0.83 (−1.30 to −0.44)	0.008
Discharge head circumference (cm)	32.0 (31.0–34.0)	31.65 (30.5–35.0)	0.67
Discharge head circumference z-score	−0.25 (−0.93 to 0.40)	−0.54 (−1.20 to 0.15)	0.15
Delta z-score for head circumference	−0.21 (−0.76 to 0.35)	0.15 (−0.72 to 0.71)	0.34
Discharge length (cm)	46.8 (45.0–49.0)	45.4 (43.2–49.5)	0.10
Discharge length z-score	−0.28 (−0.84 to 0.52)	−1.00 (−1.27 to −0.12)	<0.001
Delta z-score for length	−0.54 (−1.16 to 0.10)	−1.04 (−1.75 to −0.51)	<0.001
Necrotizing Enterocolitis rate	1 (0.4%)	0 (0%)	1.00
Intraventricular Hemorrhage rate	9 (3.6%)	3 (7.1%)	0.39
Bronchopulmonary dysplasia rate	15 (5.9%)	7 (16.7%)	0.01
Retinopathy of Prematurity rate	56 (22.1%)	32 (76.2%)	<0.001
Sepsis rate	4 (1.6%)	9 (21.4%)	<0.001

Values expressed in frequency (%) or medians and IQR. Rates calculated from cases/study population.

Of these, 101 infants with very low birth weight (VLBW) were analyzed separately (Table 3). Those that achieved target enteral feeding volume faster were slightly older and bigger, required fewer PN days, had higher growth velocity, significantly improved delta z-scores for weight and length and were discharged earlier. They also had significantly lower rates of sepsis compared to those that took longer to achieve target feeding volumes. Moreover, infants that took longer to achieve target enteral feeding volumes had 15.45 (95% CI 1.82–131.40, *p* = 0.001) higher odds of being diagnosed with sepsis during the hospital stay.

**Table 3:** Very Low birth weight infants that achieved target enteral feeding volume during the first week of life.

Variable	<8 days	≥8 days	*p*-value
# of infants	65	36	–
Birth gestational age	30 (29–32)	28 (27–30)	<0.001
Birth weight (g)	1,320 (1,220–1,450)	1,160 (955–1,322)	<0.001
Birth weight percentile	37.43% (14.42%–51.85%)	40.03% (19.98%–59.38%)	0.14
Birth weight z-score	−0.28 (−0.86 to 0.29)	−0.12 (−0.71 to 0.45)	0.21
Birth head circumference (cm)	27 (26–28)	26 (24–27)	<0.001
Birth head circumference z-score	−0.69 (−1.24 to −0.09)	−0.34 (−1.11 to 0.25)	0.33
Birth length (cm)	39.4 (38.6–40.6)	38.1 (35.6–39.3)	<0.001
Birth length z-score	0.04 (−0.86 to 0.48)	0.40 (−0.57 to 0.69)	0.22
First enteral feed in days (IQR)	1 (1–1)	1 (1–1)	0.053
First target enteral feed in days (IQR)	6 (5–8)	10 (9–11)	<0.001
First fortification in days (IQR)	6 (5–8)	11 (10–12)	<0.001
Return to birth weight in days (IQR)	12 (8.75–16)	11 (9–16)	0.98
Parenteral nutrition in days (IQR)	6 (5–8)	10 (9–12)	<0.001
Growth velocity in g/kg/day (IQR)	24.14 (20.95–27.89)	22.38 (19.20–25.30)	0.02
Length of stay in days (IQR)	47 (33–61)	58 (45.5–75.5)	0.008
Human milk (% total volume given)	77% (50.5%–100.0%)	75.5% (52.3%–90.5%)	0.71
Mother's milk (% total volume given)	46% (1%–88%)	33.5% (1.3%–81.5%)	0.54
Weight 36 weeks cGA (g)	2,187 (1,916.8–2,499.5)	2,138 (1,974.5–2,298)	0.61
Weight 36 weeks cGA z-score	−1.41 (−2.07 to −0.78)	−1.53 (−1.85 to −1.25)	0.49
Discharge weight (g)	2,379 (2,065.5–2,932)	2,237.5 (2,079.3–3,050)	0.59
Discharge weight percentile	19.72% (7.16%–34.26%)	16.45% (8.38%–26.87%)	0.62
Discharge weight z-score	−0.85 (−1.46 to −0.41)	−0.98 (−1.38 to −0.62)	0.62
Delta z-score for weight	−0.62 (−0.91 to −0.20)	−0.83 (−1.31 to −0.44)	0.01
Discharge head circumference (cm)	32 (30.5–34)	31.7 (30.5–34.98)	0.81
Discharge head circumference z-score	−0.44 (−1.23 to 0.16)	−0.56 (−1.13 to 0.10)	0.94
Delta z-score for head circumference	0.01 (−0.62 to 0.61)	0.16 (−0.70 to 0.68)	0.82
Discharge length (cm)	45.7 (44.5–48.4)	44.8 (43.2–49.1)	0.29
Discharge length z-score	−0.69 (−1.27 to −0.22)	−1.03 (−1.24 to −0.54)	0.30
Delta z-score for length	−0.49 (−1.21 to −0.07)	−0.98 (−1.69 to −0.52)	0.01
Necrotizing enterocolitis rate	1 (1.5%)	0 (0%)	1.00
Intraventricular Hemorrhage rate	6 (9.2%)	3 (8.3%)	1.00
Bronchopulmonary dysplasia rate	12 (18.5%)	7 (19.4%)	0.90
Retinopathy of Prematurity rate	39 (60.0%)	28 (77.8%)	0.07
Sepsis rate	1 (1.5%)	7 (19.4%)	0.003

Values expressed in frequency (%) or medians and IQR. Rates calculated from cases/study population.

Overall, infants who received fortification earlier had a significantly higher growth velocity, weight z-score at 36 weeks cGA, and the delta z-scores were significantly improved for weight, length and head circumference compared to their counterparts. Additionally, after controlling for birth weight and GA, those that started fortification later had significantly higher odds of sepsis compared to their counterparts; 14.13 (95% CI 2.90–68.88, *p* = 0.001). Lastly, VLBW infants who received fortification during the first week of life, had significantly higher weight growth velocity, improved delta z-scores for weight and length and lower sepsis rates compared to their counterparts.

## Discussion

In our cohort of VLBW and LBW infants, early initiation of feeding paired with faster volume advancement and earlier fortification resulted in improved z-scores for weight, length, and head circumference at NICU discharge. As preterm infants are fed earlier, they require fewer PN days and have lower rates of sepsis. Among VLBW infants, the rates of NEC, IVH, BPD and ROP did not change between the groups.

### Early initiation and rapid advancement of enteral feeds

Human milk continues to be the standard of care for feeding newborn infants, especially for those born preterm (22). Multiple studies have shown that early initiation of enteral feedings protects the infant against infection and is beneficial for improving feeding tolerance (11, 23). Negative biological changes have been described in relation to the intestinal barrier as well as to the microbiome of the preterm infant when feeds are held for a prolonged time (11, 13). On the other hand, earlier initiation of feeds has been shown to be protective with no consequential increase in NEC rates or other complications, and to have possible improvement of common neonatal comorbidities like sepsis (10, 11, 13, 23–26). Also, studies have described preterm infants tolerating the start of enteral feeds at full volume on day of life 1 (60–80 cc/kg/day) with no increase in comorbidities (27, 28). However, there is no data showing a clear long-term benefit of this practice.

A relevant and frequently discussed topic continues to be that of trophic and enteral feeding volume advancement. Current evidence suggests safe advancement at rates of 30cc/kg/day with no data supporting an extended (more than 24 h) period of trophic feeding volume (9, 23). Existing recommendations suggest starting feedings within the first 72 h of life at trophic volume and then advancing as soon as possible by rates of 20–30 cc/kg/day until achieving full enteral feeds (13, 29). However, this recommendation should be considered the minimal requirement for feeding preterm infants, and faster advancement should be advised for those infants that demonstrate adequate tolerance of enteral feeds. As described by others, infants at our institution who achieved target enteral feed volumes faster, were associated with lower rates of sepsis; this was also noted in VLBW which represent a more vulnerable population (11, 30).

### Fortification of preterm enteral feeds

Uncertainty remains as to whether to focus on feeding advancement or earlier fortification of enteral feeds (31). Wynter et al. (32) randomized 52 preterm infants to early (bovine based HMF to 22 kcal/oz upon initial feeding) or late (at 10 days of age) fortification and did not find statistically significant differences, though, infants tolerated the intervention well with no adverse effects and achieved target enteral feed volumes by 12 DOL. Salas et al. (33) randomized 150 infants to receive early (human based fortification to 24 kcal/oz since day 1) or later fortification with bovine-based HMF at DOL 14, with full enteral feeds achieved by DOL 8. They found non-significant improvements in growth parameters and what's more, infants tolerated the intervention well.

As shown in our cohort, starting fortification during the first week of life significantly improved the z-score discharge parameters compared to those who did not. Moreover, for every additional day that fortification is delayed, the odds of having a delta z-score for length ≥0 decreases (OR = 0.2, 95% CI 0.85–0.99, *p* = 0.03); consequently, the risk of the infant developing growth failure (delta z-score ≥1.2) for length (OR = 0.92, 95% CI 0.85–0.99, *p* = 0.03) increases. Of note, infants fed earlier had improved delta z-scores for weight and length, while adding fortification during the first week of life significantly impacted the delta z-score for head circumference in addition to the weight and length. As a result, these infants demonstrate improved neurodevelopmental outcomes in association with adequate head circumference growth, a crucial finding (3, 33, 34). These improvements of growth parameters as a result of early fortification have been demonstrated to continue even after NICU discharge (26).

In our cohort of VLBW infants, we observed that infants fed faster had significant improvements in the rates of sepsis with no changes in the rates of NEC, IVH, BPD and ROP. Moreover, for every additional day to achieve target volume enteral feeds, the odds of being diagnosed with sepsis increases by 1.45 (95% CI 1.13–1.86, *p* = 0.003). Consistent with recent publications, sepsis is higher when there is a delay in achievement of target enteral feed volumes and with the ensuing increase in PN use (26, 30, 35). Additionally, delaying advancement of enteral feeds has been associated with increased total fluid intake which can have negative consequences in the preterm infant (36).

Human milk use has been associated with improved NEC and sepsis rates, especially MOM; however, availability of MOM continues to be an issue (16). In recent years, our unit has become stricter in transitioning from DBM to preterm formula once at 34–35 weeks cGA if MOM supply is insufficient. This led to a decreased use of human milk during the infant's entire hospitalization, specifically MOM; yet, the practice did not affect the rates of sepsis or NEC in both cohorts (all preterm infants and those VLBW).

The definition of postnatal suboptimal growth, extra-uterine growth restriction or postnatal growth failure continues to be a topic of controversy (37, 38); however, it is known that infants who do not meet desired growth are at risk of adverse outcomes later in life (3, 4, 34, 38). Furthermore, high rates of postnatal suboptimal growth have been reported in NICUs around the world (1, 39), suggesting a global impact. In our analysis, we found that malnutrition (decline in delta z-score >1.2) was diagnosed in 35 (11.7%) infants, which is lower than reported by others (1, 39). Even decreases of >1SD in delta z-scores for weight and HC have been associated with adverse neurodevelopmental outcomes (38). We found that 62 and 42 of infants in our cohort had changes >1 of their delta z-scores for weight and HC respectively.

In our VLBW population, for every additional day needed to achieve target volume enteral feeds, the odds of being diagnosed with malnutrition increased by 1.25 (95% CI 1.04–1.49, *p* = 0.02). Kakatsaki et al. described that nutritional factors like day of initiation, attainment of full enteral feeds and the duration of PN were associated factors to postnatal growth failure (39). Growth improvements have also been reported in the literature when feeding guidelines are implemented and growth/nutrition are closely followed in preterm infants (6, 15) with positive impacts that persist after NICU discharge (3, 10, 26).

Limitations of our study include: a) its retrospective nature; b) the lack of a larger population of infants born less than 28 weeks GA or Extremely Low Birth Weight (ELBW), which constitutes a more vulnerable population; c) the lack of categorization of the infant's clinical status at admission and, c) neurodevelopmental follow up information after NICU discharge. Since our population included mostly VLBW and LBW infants, these findings need to be taken with caution when treating ELBW infants.

## Conclusion

Early initiation, faster advancement and earlier fortification of enteral feeds are associated with improved delta z-scores for weight, length, and head circumference in preterm infants. These changes correlate with decreased PN use and rates of sepsis with no changes in the prevalence of NEC. Providing only early fortification in order to improve growth failure is not supported by the literature (32, 33); therefore, efforts should be made to advance enteral feeding as soon as possible so PN and central line use can be discontinued and fortification of enteral feeds can be initiated (26). Currently, there is no adequate evidence to support enteral fasting during ductus arteriosus treatment or blood transfusions (10). Feeding guidelines need to be developed per each individual institution as they improve nutritional outcomes (10, 12, 40). Preterm infant growth needs to be strictly monitored to ensure their development and prevent postnatal suboptimal growth (30, 39).

## Data Availability

The raw data supporting the conclusions of this article will be made available by the authors, without undue reservation.
